# Dengue haemorrhagic fever in chronic kidney disease and heart failure: challenges in fluid management

**DOI:** 10.1186/s41182-024-00600-9

**Published:** 2024-04-24

**Authors:** Manudi Vidanapathirana

**Affiliations:** Professorial Medical Unit, National Hospital of Sri Lanka, Colombo, Sri Lanka

**Keywords:** Dengue haemorrhagic fever, Heart failure, Chronic kidney disease, Fluid management

## Abstract

Dengue haemorrhagic fever (DHF) is recognized to have high mortality in patients with chronic kidney disease (CKD) and heart failure (HF). They are at high risk of shock during the ascending limb of the critical phase of DHF, fluid overload during convalescence and bleeding throughout the entire illness. Physiological changes and medications used in CKD/HF make the diagnosis and monitoring of DHF difficult. Treatment with standard fluid regimens also poses a challenge due to the propensity for fluid overload. As a result, standard dengue guidelines do not provide recommendations on fluid management regimens in DHF with CKD/HF. This article provides a narrative review on the existing evidence for management of DHF in patients with volume-changed states such as HF, CKD and nephrotic/ nephritic syndromes. It will explore the relevant diagnostic and therapeutic dilemmas, acknowledge the challenges for developing guidelines and recommend strategies to improve fluid management in these groups of patients.

## Introduction

Dengue haemorrhagic fever (DHF) is a severe presentation of dengue, characterized by plasma leakage and, at times, haemorrhagic manifestations [1]. The plasma leakage occurs during a 48-h period known as the critical phase, and it is evidenced by a rise in haematocrit of at least 20% [1, 2]. Critical phase usually starts approximately 3–5 days after onset of fever [1]. The critical phase consists of an initial 24-h period called the ascending limb, during which the rate of plasma leakage may gradually increase, and a subsequent 24-h period, during which the rate of leakage gradually declines [1]. This is followed by a convalescent period characterized by plasma reabsorption and return to homeostasis [1]. The concern during the critical phase is intravascular volume depletion and shock, and the concern during the convalescence phase is fluid overload [1, 2]. Haemorrhagic manifestations may occur at any point of the febrile illness in DHF [1].

Standard guidelines on the management of DHF highlight the need for adhering to precisely calculated fluid regimens [1–5]. The generally recommended fluid quota for 48 h in DHF is the maintenance fluid combined with 5% of deficit, which usually amounts to 4600 millilitres in an individual weighing 50 kg or above [1]. The need for such strict fluid regimens in DHF is for the purpose of striking a careful balance between prevention of shock in the leaking phase and prevention of fluid overload in the convalescence phase [2]. Existing guidelines provide recommendations on the fluid quota and the rate of fluid administration to be followed in healthy individuals with DHF during the critical phase. However, no guideline explores the fluid administration to be followed in DHF in patients with changed intra- and extravascular volume states, such as in heart failure (HF) or chronic kidney disease (CKD). Existing guidelines acknowledge that patients with HF and CKD are in the high-risk category and must be admitted to receive in-patient fluid management, but there is no further guidance given in this regard.

Fluid administration in volume-changed states is problematic due to two reasons. Firstly, CKD and HF are both conditions that are prone to fluid overload, in the form of pulmonary oedema and systemic venous congestion [6]. This risk is higher with anuric or oliguric patients with CKD. When these individuals are treated with the same fluid regimens as those used for DHF in healthy individuals, they are at high risk of developing fluid overload. Secondly, despite the risk of fluid overload, these individuals are still at risk of shock during the leaking phase, if an adequate fluid quota is not administered. Naturally, the question arises as to how fluid administration should be guided in these patients to achieve this delicate balance.

This article provides a narrative review on the existing evidence for management of DHF in patients with volume-changed states such as CKD, nephrotic and nephritic syndromes and HF. It will explore the relevant diagnostic and therapeutic dilemmas, acknowledge the challenges for developing guidelines and recommend certain strategies to improve fluid management in these groups of patients.

## Methods

A literature search was conducted on the topic using Google Scholar and Medline from February–March 2024. The following Boolean operators were used to focus the search, and only articles in English were included.

i) "dengue haemorrhagic fever" AND "chronic kidney disease" NOT "acute-kidney-injury"
ii) “dengue haemorrhagic fever” AND “heart failure”

For the first search item, there were 361 results. Duplicated articles, irrelevant articles, articles on acute kidney injury (AKI) secondary to dengue and articles on dengue in renal transplant recipients were excluded. After exclusion, 13 articles were left for analysis.

The second search item yielded 1660 results. Duplicated articles, irrelevant articles, articles on heart failure/ myocarditis/ cardiomyopathy secondary to dengue and articles without a focus on fluid management were excluded. After exclusion, 1 article was included in the review.

## Implications of CKD and HF on the pathophysiology of DHF

Plasma leakage in DHF occurs due to increased capillary permeability secondary to virus-related immune dysregulation [2]. Antibody-dependent viral enhancement is a recognized mechanism which increases the likelihood of plasma leakage and occurrence of DHF, in a secondary infection [7]. Once the virus enters macrophages facilitated by previously formed antibodies, a cytokine cascade comprising of interleukins comes in to play [7]. This is responsible for endothelial injury and vascular permeability that consequently results in leakage.

Cytokines such as tumour necrosis factor (TNF)-alpha and interleukin (IL)-6 are responsible for endothelial injury in DHF, and these are reported to be increased in patients with CKD [8]. This increase in cytokines, combined with the intrinsic uraemic milieu of CKD which further aggravates endothelial dysfunction, can increase the risk and amount of DHF-related plasma leakage in CKD [8]. Additionally, uraemia in CKD reduces thrombogenecity through dysfunction of platelets and von Willebrand factor [8]. This can in turn increase the haemorrhagic manifestations of DHF in patients with CKD.

A study carried out by Lee et al. in 2019 identified that impaired immunological mechanisms occur in end-stage renal disease (ESRD) patients with dengue [9]. It was found that levels of several cytokines, including IL-8, IL-10, IL-12p40, TNF-α, monocyte chemoattractant protein 1, vascular endothelial growth factor and granulocyte macrophage colony-stimulating factor, were significantly lower in the ESRD population than in the control group. Viral load cycle threshold values were also significantly lower in the ESRD group at 6 h and 24 h post-infection. No significant difference in the viral load cycle threshold values between the two groups was found at 48 h and 72 h post-infection. However, this was conducted as an in vitro study, and its translation into clinical practice, in terms of affecting the clinical progression of DHF in CKD, is not very clear.

## Increased mortality in DHF with heart disease and CKD

Several studies conducted among dengue patients, both with and without plasma leakage, have concluded that mortality in those with heart disease and CKD is higher compared to healthy controls [10]. Reasons for the increased mortality in these groups may be manifold, such as propensity for haemodynamic fluctuations, risk of bleeding and use of drugs such as anti-hypertensives, diuretics and antiplatelet agents that can worsen complications in dengue [11, 12]. Improper fluid management regimens may also contribute to increased mortality in these groups, and it may be an important modifiable factor that can be optimized, to target a reduction in mortality.

A study done by Lee et al. published in 2023 assessed characteristics of 138 dengue patients with CKD [11]. This study included patients with and without plasma leakage and demonstrated a high mortality rate of 46.3% (n = 64) among dengue patients with CKD. The stages of CKD of the patients who died were stages 2 and 3 in 34.4%(n = 22), stages 4 and 5 in 51.5% (n = 33) and end-stage renal disease on dialysis in (4.1% (n = 9)). However, the group consisting of patients with ESRD was underrepresented. There were 57 (41.3%) and 58 patients (42.02%), respectively, in the groups for CKD stages 2–3 and stages 4–5, but only 23 (16.7%) patients for the group containing patients with ESRD. The underrepresentation of ESRD patients in this study may have accounted for the seemingly lower mortality seen in the ESRD group. When fatality is calculated for each group, deaths comprised 38.5% in CKD stages 2–3, 56.9% in stages 4–5 and 39.13% in ESRD. Complications experienced by the patient that died in the CKD group were gastrointestinal bleeding (56.3% (n = 36)), severe hepatitis (40.6% (n = 26)), pneumonia (25%(n = 16)), pulmonary oedema (12.5%(n = 8)) and bacteraemia (9.4% (n = 6)). The median time from symptom onset to death was 6 days. Risk factors associated with high mortality were altered level of consciousness at presentation, pulmonary oedema, leucocytosis and severe hepatitis.

Another study in 2023 corroborates the increased mortality seen in dengue patients with CKD [12]. This study included 433, 802 dengue patients, both with and without plasma leakage. Of the total number, 0.5% (n = 2134) had a diagnosis of CKD. Details on the stages of CKD in these patients were not given. Characteristics observed more commonly in the CKD group were tachycardia, haemodynamic fluctuations, increased capillary refill time, pulmonary oedema with respiratory failure and higher rates of severe bleeding (e.g. haematemesis).

Both studies described above show that CKD patients with dengue were more at risk of haemodynamic fluctuations and gastrointestinal bleeding. While only the latter study comments on the occurrence of intravascular volume depletion, both studies have noted an increased occurrence of pulmonary oedema in the CKD group.

With regard to heart disease, a study published in 2017 has recognized heart disease as an independent predictor of severe dengue and severe organ involvement in dengue [13]. Mechanisms hypothesized for this these adverse manifestations are endothelial dysfunction and increased nitrous oxide contributing to the cytokine storm and increased vascular permeability. It is recognized that renal autoregulation is offset, if cardiac output in a patient already having heart disease/ heart failure is further compromised during dengue, thus increasing the risk of kidney injury.

### Diagnostic and therapeutic challenges of DHF in CKD and heart disease

Factors that distinguish DHF from uncomplicated dengue are presence of capillary leakage resulting in extravascular fluid accumulation, development of extensive petechae/ ecchymosis, overt bleeding, marked thrombocytopenia, hypoalbuminemia, elevated liver enzymes and hyponatremia [8]. Several of these factors are seen in CKD patients as part of CKD itself and therefore may not help to characterize the severity of dengue in this population. Thrombocytopenia is seen in CKD patients due to uraemia-related marrow suppression, or heparin-induced thrombocytopenia [8]. Petechae may be seen in CKD patients due to inherent thrombocytopenia, thrombasthenia or increased vascular fragility [8]. Hyponatremia is seen in CKD due to dilutional hyponatremia in hypervolaemic states, and hypoalbuminemia is seen in CKD due to an imbalance between synthesis and loss [14, 15].

A rise in haematocrit is used in dengue management to identify the onset of leakage in DHF [2]. However, CKD carries with it certain confounders that limit the utility of haematocrit as a useful marker of leakage in this population. A rise in haematocrit unrelated to leaking may be seen in these patients due to the use of diuretics with a restrictive fluid regimen, for fear of causing fluid overload [16, 17]. This may lead to a false diagnosis of DHF. Additionally, as reiterated above, CKD patients are at high risk of bleeding during DHF, which may mask a rise in haematocrit and may lead to leakage being overlooked [17].

A study carried out by Thomas et al. in 2018 among renal transplant recipients and patients with CKD showed that there was increased haemoconcentration among the CKD group, compared to the transplant group and control group, with normal renal functions [18]. It is postulated that reluctance for fluid administration in the CKD group may have accounted for this finding.

Nadir WBC count was lowest in renal transplant recipients when compared to CKD patients and control group, hypothesized to be due to bone marrow suppression due to the use of mycofenolate mofetil and azathioprine in their immunosuppressive regime. This may also account for the longer time taken for normalization of platelet count in renal transplant recipients. Occurrence of bleeding manifestations was seen in a total of 7 patients across all groups and was not statistically different between the 3 groups.

A study carried out by Chen et al. in 2019 identified certain other difficulties associated with diagnosis and treatment of dengue in ESRD patients on haemodialysis [19]. One reported diagnostic problem was dismissal of symptoms like vomiting and abdominal pain, as markers of severe dengue, since these symptoms which are common among ESRD patients on dialysis were likely to be attributed to uraemia-/ dialysis-related symptoms. Treatment problems encountered were the development of hypervolaemia and pulmonary oedema, with the use of the fluid regimen recommended by World Health Organization, and the increased occurrence of intradialytic hypotension. Expectedly, bleeding manifestations were more frequently noted.

According to standard dengue guidelines, acidosis, bleeding, hypocalcaemia and hypoglycaemia are factors that cause refractory shock in DHF and lead to the development of dengue shock syndrome (DSS, 1). All four factors occur more commonly in CKD patients, due to CKD itself, and this must be borne in mind treated accordingly, in CKD patients with shock in dengue.

No studies have commented on the limitations of individual vital parameters in monitoring of DHF in CKD, but these can be anticipated from the physiological changes that occur in CKD. Blood pressure which is high CKD due to arterial calcification and volume overload may be maintained until significant capillary leakage has taken place in DHF and therefore may be an insensitive marker of intravascular volume depletion [20]. Urine output too is a poor marker due to the oliguric/ anuric state exhibited by some patients with CKD.

There are no studies focused on the diagnostic and therapeutic dilemmas among patients with heart failure in dengue, but some challenges can be estimated from the physiological changes occurring in HF. Factors confounding the diagnosis of DHF in heart failure are the baseline existence of pleural effusions and ascites in congestive cardiac failure and the baseline presence of hypoalbuminemia and hypercholesterolemia [21, 22]. Haematocrit may be misleading in heart failure, due to the use of diuretics, fluid restriction as part of management in heart failure, and due to increased risk of bleeding with dengue due to antiplatelet use. Vital parameters such as pulse rate, blood pressure and pulse pressure may be misleading depending on the patient’s baseline ejection fraction and use of medications altering these parameters [23]. The same applies for urine output.

Various trials have demonstrated the limited clinical utility of haemodynamic parameters such as central venous pressure and pulmonary capillary wedge pressure in guiding fluid responsiveness in any condition requiring fluid resuscitation [24]. This is primarily due to the varying effects on these parameters by cardiac contractility. As a result, dynamic parameters such as stroke volume variation, pulse pressure variation and change in vena cava diameter have been proposed to guide fluid therapy. Due to the lack of superiority of a single variable in clinical studies, a combination of these is recommended in the context of the clinical picture. However, further assessment of the utility of these parameters in DHF is needed before recommendations can be made.

Use of inferior vena cava (IVC) collapsibility has been shown to correlate with the rise in haematocrit in DHF and dengue shock syndrome, and the use of IVC collapsibility has therefore been reported to be a better marker for guiding fluid therapy than haematocrit [25]. This may be especially true in instances when haematocrit may misleadingly not show a rise, due to combined leaking and bleeding. The use of the sum of pleural and peritoneal fluid volumes is not recommended to be used to guide fluid therapy, since it is likely to represent an underestimation of the true value [26].

In terms of fluid administration, the battle between crystalloids and colloids remains age old [24]. For healthy individuals that develop DHF, it is recommended to use crystalloids for fluid management and reserve the use of colloids for certain special instances [2]. Colloids are indicated in DHF when there is persistent shock despite the use of two crystalloid boluses, shock in the descending limb of the critical phase with features of fluid overload and signs of shock with near completion of the fluid quota [2]. However, the use of 0.9% saline, which is the most commonly used crystalloid, is known to be associated with hyperchloraemic metabolic acidosis [24]. This can worsen the pre-existing metabolic acidosis in renal failure and result in detrimental consequences such as renal vasoconstriction, further decrease in glomerular filtration rate and negative inotropy [24]. There is currently no research available to recommend alternate crystalloids or colloids over the use of 0.9% saline in dengue in patients with CKD [24]. The use of crystalloids therefore remains the standard of practice [24]. A randomized controlled trial carried out among 230 Vietnamese children with DSS has shown that Ringer Lactate (RL) had the longest recovery time, compared to normal saline, gelatin and dextran [27]. There is a theoretical risk of worsening hyperkalaemia and acidosis with RL in CKD patients due to its constituents [27]. This makes it a questionable choice for this population. While colloids have been shown to perform better in shock states in some studies, the ‘reverse osmotic’ effect where the osmotically active colloid leaks out and worsens capillary leakage needs to be considered [27]. If as hypothesized earlier, vascular endothelial dysfunction and risk of leakage are in fact higher in the CKD population, the author infers that colloids may not be a suitable intravenous fluid for this population [8].

### Case reports on the fluid management of DHF in volume-changed states

There are 4 case reports/series which focus on fluid management of DHF in CKD [16, 17, 28, 29].

A case series by Kuo et al. outlines the progression of 3 patients with CKD who developed DHF and proceeded DSS and death [28]. Problems with diagnosis existed for all 3 patients since the suspicion of dengue had been low, and initial symptoms had been attributed to uraemia. The diagnosis had only been entertained later and proven serologically. All 3 patients had also developed acute liver injury and gastrointestinal bleeding, as part of DSS. The fluid regimen followed in these 3 patients is not given in the case series, but it could possibly be presumed that since the patients developed DSS, there might have been initial under-filling, due to the general restrictive intake of CKD. Under-replacement of fluids may have continued for a significant duration of the illness due to low suspicion of dengue. All 3 patients had also received blood products in the form of fresh-frozen plasma (FFP) and red cell concentrate. It is possible that transfusion of FFP to these patients with CKD may have served as a risk factor for overfilling and pulmonary oedema during the convalescence phase. Only one patient had been dialyzed, and the risks of worsening haemodynamic stability in an already unstable patient are highlighted through this. While this case series does not reveal much in terms of fluid management, which is the focus of the current review, it is useful since it draws attention to maintaining vigilance for dengue in CKD patients in high-prevalence areas and showcases the detrimental one-way path that ensues when DHF is not recognized and managed properly. On another note, these patients had received desmopressin as a treatment for bleeding. Whether desmopressin is useful in terms of preventing or treating bleeding in dengue with CKD needs more research, and its unpredictable effects in terms of water retention, and potential for fluid overload in the descending limb and convalescent stage make its use questionable.

Another case report by Lim et al. in 2019 outlines the progression of a patient with ESRD developed DF and had signs of intravascular volume depletion at the time of admission [16]. When he reached a cumulative intake of 2500 ml, he started showing signs of leakage, with development of pleural effusions. Regular haemodialysis was continued, with the ultra-filtrate being guided by volume status and phase of dengue. The ultra-filtrate has had to be increased during the recovery phase, although the exact volumes are not disclosed. The fluid regime that was used was 0.35 mL/kg/h (500 mL) of 0.9% saline over 24 h, 0.9% l saline 3 mL/kg/h (180 mL/h) for 4 h, 0.9% saline 1 mL/kg/h (60 mL/h) for 3 h, then off drip to encourage oral intake of 50–100 mL hourly. It was planned to convert to colloids if intravascular hypovolemia persisted or consider blood transfusion if signs of overt bleeding developed, but neither was required in this patient. Clinical assessment of fluid status was done by regular monitoring of vital signs, charting of input output and assessment of skin turgor and mucosa. Investigations that were used for assessment of volume status were haematocrit, ultrasonic assessment of inferior vena caval collapsibility and ultrasonic assessment of the third spaces to detect pleural effusion and ascites. The article comments on other available invasive methods such as monitoring of central venous pressure, central venous oxygen saturation, cardiac output, pulmonary arterial pressure, mixed venous oxygen saturation and stroke volume variation, which may be required if a patient becomes critically ill requiring micromanagement of fluid and tissue perfusion, but appears to not have used them for the patient reported. This article also recommends temporarily withholding antihypertensive therapy and restarting them during the recovery phase.

Additionally, it makes recommendations on practicing heparin-free dialysis until the platelet count rises to 100, stopping blood thinning agents when the platelet count drops below 100 and temporarily stopping azathioprine/ mycophenolate mofetil due to their myelosuppresive effects. It recommends to double the dose of steroids to prevent Addisonian crisis, in those who are on long-term steroids.

A case report from Sri Lanka outlines the case of a patient with CKD stage 3A due to lupus nephritis [17]. The patient has presented with nephrotic syndrome and subsequently developed DHF complicated with bleeding and fluid overload. The diagnostic challenges in this case were recognition of DF in a patient with lupus-related cytopenias and identification of leakage in a patient with effusions and ascites due to nephrotic syndrome. The main management challenge was determining the amount and rate of fluid administration. Due to the presence of baseline lupus-related cytopenias, platelet counts could not be used as a marker of severe dengue in this patient. The baseline presence of extravascular fluid in the form of pleural effusions and ascites, and the presence of per vaginal bleeding obscuring a haematocrit rise, made it difficult to diagnose leakage in this patient. In her case, DF was diagnosed with the use of NS1 antigen which is more than 99% specific for dengue, and leakage was diagnosed with the timing of transition from febrile to afebrile status without clinical improvement and tenderness in the right hypochondrium. Fluid management followed a restrictive protocol. However, the total fluid quota of 4600 ml calculated for her ideal body weight was theoretically allowed, in case features of shock developed. Furosemide and albumin which were part of the management of nephrotic syndrome in this patient were continued during the critical phase. A total amount of 3150 ml of crystalloid fluid was used during the critical phase and she did not develop signs of shock. However, despite the use of a restrictive fluid regime and the continuation of diuretics, she developed fluid overload during the descending limb. This required intubation with mechanical ventilation and continuous renal replacement therapy.

A case report from India published in 2024 describes the challenging case of a 16-year-old female with ESRD on regular haemodialysis, who developed dengue fever [29]. The dengue fever was serologically confirmed. This case was complicated by the rapid development acute respiratory distress syndrome (ARDS) which intensified the already existent challenges of fluid management. It is unclear from the case whether the patient had uncomplicated dengue or DHF. There is mention of bilateral pulmonary shadows with minimal pleural effusions on a chest x-ray, but this could have been accounted for by ARDS. A point-of-care ultrasound has also been performed, but there is no comment on the presence or absence of pleural effusions or ascites. Fluid therapy in this patient has been guided by IVC collapsibility index. Although the exact amounts of fluid used are not mentioned, sufficient intravenous fluid has been administered to reduce the IVC collapsibility index from more than 50% to less than 30%. Intravenous crystalloids had been used for resuscitation. There are two factors that have complicated fluid management in this case, apart from the challenge imposed by ESRD. First is that, there appears to have been additional sepsis confounding the clinical picture. The patient has had dysuria and right sided flank pain, possibly indicating pyelonephritis. The white cell count has been 30,000/mm^3^, which also favours a diagnosis of bacterial sepsis. In fact, the authors themselves have managed the patient for sepsis with intravenous antibiotics and inotropic support. Therefore, the fluid requirements in this case may have been increased by the presence of sepsis. The second additional challenge to fluid management is the development of ARDS. ARDS may worsen the burden of fluid overload due to the migration of fluid into the alveoli. It is thus a dilemma in this case to decide on the approach to fluid therapy. While the exact amounts of fluid given to this patient are not disclosed in the final report, the authors suggest a protocol of goal directed resuscitation during the shock phase, followed by a more restrictive protocol once shock has resolved. This patient has also been given sustained low efficiency dialysis in an ICU, but has succumbed to the illness. The authors recommend to use both IVC parameters in the form of diameter and collapsibility index, and point-of-care lung ultrasound to guide fluid therapy.

These published cases illustrate the importance of being vigilant of dengue and highlight the increased risk of bleeding, DSS and fluid overload that occurs in CKD patients with dengue.

Three of the cases give an insight in to the fluid regimen. It is evident from the third case that while a restrictive regimen was able to circumvent shock, it still resulted in fluid overload [17]. However, not all patients may be able to circumvent shock with a restrictive approach, and adhering steadfastly to a restrictive protocol in patients with severe leaking during the critical phase may increase the risk of shock and mortality. Accordingly, the fourth case favours a more liberal approach during the period of shock, followed by a more restrictive approach after mitigation of shock [29]. This carries with a risk of fluid overload during the descending limb and convalescence of the critical phase. The author deduces from this that the fluid quota would need to change in patients with CKD, but hourly administration rates need to take in to account the real-time risk of shock versus overload.

There is only one published study on fluid management of DHF in heart failure [30].

A case report from Malaysia describes the management of dengue, without capillary leakage in a patient with heart failure [30]. The ejection fraction of the patient was not included in the report. Dengue management comprised of input output monitoring, daily input of 1 L of fluid and continuation of oral furosemide 40 mg daily. The plan was to continue the same fluid regime if the urine output was more than 500 ml per day and weight gain per day was 1 kg or less. However, it is mentioned that the patient had fluid losses in the form of vomiting and diarrhoea initially.

It is difficult to glean information that can be synthesized into recommendations from this case since it did not have plasma leakage. However, it is reasonable to speculate that an alternate, less-restrictive fluid regimen may have been required in case of plasma leakage. The patient was on diuretics and had volume losses in the form of vomiting and diarrhoea, which if combined with plasma leakage may have resulted in shock and DSS, if continued on a restrictive regimen.

### Challenges for establishing guidelines and strategies proposed

The main problem of management of DHF in the volume-shifted state is the difficulty in striking the balance between adequate filling to prevent shock and avoidance of overfilling. Additional factors interfering with this balance are the use of medications such as anti-hypertensive medications and diuretics, and the increased risk of bleeding. The author recognizes the difficulty in having a generic guideline for this population, due to the individual variations in the degree of volume shifting, differences in types and doses of medications used and oliguric/anuric state seen only in some patients.

In spite of these factors, the author proposes the following strategies to better guide fluid management in this population:

1. Maintenance of vigilance for dengue and DHF in high prevalence areas. One must be careful not to attribute certain symptoms such as vomiting and abdominal pain to uraemia alone, if associated with fever.
2. Involvement of a multidisciplinary team with physicians, infectious disease specialists, nephrologists, cardiologists, intensivists and transfusion physicians was relevant for management of DHF in CKD and HF.
3. Recognition of the limitations of diagnosis of plasma leakage in DHF in CKD and HF.

Pleural effusions, ascites, rise in haematocrit, hypoalbuminemia and hypercholesterolemia may not be useful in detecting leakage since these may already exist at baseline in patients with CKD and HF. Therefore, other markers recommended in standard guidelines may have to be relied upon e.g. settling of fever with a platelet drop to less than 1,000,000/μL, tenderness in the right hypochondrium and clinical deterioration with settling of fever [2].

4. Recognizing that the guideline recommended fluid quota might be harmful for patients with HF/CKD and deciding on an individual fluid quota using the clinicians’ judgement of risk of shock versus fluid overload
5. Use of all vital parameters in context of each other and the clinical picture during DHF monitoring, due to the following limitations:
6. Pulse rate: Inability to mount a tachycardia due to the use of beta blockers
7. Blood pressure and pulse pressure: Low baseline blood pressure and pulse pressure in heart failure due to low ejection fraction, maintenance of systolic blood pressure till the last stages of shock in CKD due to high baseline blood pressure and arterial calcification
8. Urine output: Misleading urine output to the use of diuretics and the inherent oliguric/anuric nature
9. Vital parameters may change due to worsening of the original comorbidity (e.g. worsening of heart failure, nephrotic syndrome) rather than due to severe dengue
10. Use of investigation markers in context of each other and the clinical picture, due to the following limitations:
11. Thrombocytopenia may be present at baseline in CKD
12. Haematocrit rise may not always be present in CKD and heart disease patients with leaking, due to high risk of co-existent bleeding
13. Haematocrit rise may be seen due to non-leaking related reasons, such as inadequate fluid administration and use of diuretics
14. Hypoalbuminemia and hypercholesterolemia, which are markers of leaking, may be seen at baseline in heart failure, CKD and nephrotic syndrome
15. Considering the use of IVC collapsibility in combination with vital parameters, haematocrit and the clinical context to guide fluid therapy
16. Guiding hourly fluid administration rates bearing the above principles in mind.

Rate of leakage in DHF generally follows the shape of an inverted V, with a rising rate in the ascending limb of the critical phase with maximal leakage at the peak and a decreasing rate in the descending limb with cessation of leakage by 48 hours [2]. Hourly fluid administration in DHF can vary from 2 to 10 millilitres (ml) per kilogram (kg) per hour (h, 2). The hourly administration rate will need to be adjusted based on the assessment of degree of leakage as indicated by vital parameters and haematocrit [2].

Stable patients will have normal vital parameters with a minimal rise in haematocrit. They can be managed with 2 ml/kg/h of fluids [2].

Unstable patients can be in pre-shock or shock [2]. Pre-shock is indicated by cold extremities, prolonged capillary refill time, tachycardia, tachypnea, narrowing of pulse pressure and postural hypotension [2]. Compensated shock is indicated by a narrowing of pulse pressure to 20 mmHg or less [2]. Decompensated shock is indicated by reduced systolic blood pressure to less than 90 mmHg or a reduction of 20% of the baseline systolic blood pressure [2]. Profound shock is indicated by undetectable blood pressure [2].

Maximal rate of fluid leakage that occurs in shock is 10 ml/kg which is the amount required by patients with shock [2]. This can be given over 1 h in compensated shock, or as a fast bolus in decompensated and profound shock [2].

A rate of administration in between 3 and 7 ml/kg can be used for patients in pre-shock, depending on the degree of derangement of vital parameters, rise in haematocrit and the response to fluids administered in the preceding hour [2]. It must be borne in mind that rapid leakage only lasts for a short time, and the requirement for fluid in between 5 and 7 ml/kg/h will be quite short [2]. After the mitigation of this period, stabilization of vital parameters and haematocrit should prompt clinicians to gradually reduce the hourly rate of fluid administration in a stepwise manner [2]. It should also be noted that all parameters should be assessed collectively, and decisions should not be based on a single parameter individually [2]. Fluid administration that is increased merely for the purpose of maintaining urine output and reducing haematocrit in patients with otherwise stable vital parameters can lead to fluid overload [2].

During the descending limb too, monitoring must continue as in the ascending limb [2]. Provided that parameters remain stable, the author suggests that reduction in fluid administration to less than 2 ml/kg/h can be attempted at the physician’s discretion to prevent fluid overload.

Renal replacement therapy in the form of sustained low efficiency dialysis or continuous renal replacement therapy may be needed during the critical phase, depending on the presence of overload and other indications for dialysis [17, 29].

The author speculates that a focus on mitigation of shock rather than fluid overload may be more important during the ascending limb of the critical phase [29]. Unresolved shock may lead to organ failures including acute liver injury with liver failure, deterioration of renal function, disseminated intravascular coagulation and death [2]. An observational study in Sri Lanka that assessed fluid requirements of patients with DHF showed that there were some DHF patients who required fluid in excess of the calculated fluid quota (more than maintenance and 5% of deficit), sometimes requiring more than 7.5% of the deficit [31]. Surprisingly, the incidence of fluid overload was not as frequent as expected, but was seen more in individuals that exceeded more than 7.5% of deficit in terms of fluid administration. The study does not comment on the comorbidities of these individuals, but presumably had been conducted among healthy individuals. Therefore, their reduced tendency for fluid overload cannot be translated in to that of the CKD or HF population. Use of such large amounts of fluid will inevitably result in fluid overload, in these special populations [6]. But this study is important in showing that some patients with DHF have severe leakage, and therefore, their fluid requirements may exceed calculated or predicted values [31]. In such patients, care must be taken with fluid management with an aim of preventing shock.

On a final note, the author emphasizes the importance of management of pre-shock and shock using the above principles, hourly monitoring with special attention to trends in vital parameters and use of clinician discretion in reducing hourly fluid administration during the descending limb and the use of renal replacement therapy when necessary.

9. Being mindful of the occurrence of acidosis, bleeding, hypocalcaemia and hypoglycaemia, which patients with CKD are prone to and may cause refractory shock
10. Considering withdrawal of anti-hypertensive medications and diuretics during the ascending limb of the leaking phase, if blood pressure is marginal
11. Considering re-initiation of diuretics during the descending limb of the leaking phase to prevent fluid overload
12. Considering withdrawal of antiplatelet medications once platelet count is below a certain level according to clinician judgement of bleeding risk versus thrombotic risk. Re-initiation can be considered once platelets are seen to be rising and above a reasonable level deemed by the managing physician.
13. Transfusion of blood according to the indications given in standard guidelines—consider transfusion under diuretic cover if fluid overload is deemed a valid risk
14. Adjustment of the ultra-filtrate in the routine dialysis prescription according to the volume status in the patient
15. Use of heparin free dialysis until the platelet count is seen to be rising and above a reasonable level as per physician discretion.

The author proposes these as suggestions for consideration, rather than recommendations, due to the individual differences in comorbidities and dengue progression.

### Gaps in research

There is further evidence required on the clinical utility of invasive modalities of haemodynamic monitoring, choice of intravenous fluid due to the problems associated with use of 0.9% saline in CKD and the clinical utility of desmopressin to treat bleeding due to its effects on intravascular volume.

## Conclusions

Diagnosis and management of DHF in CKD and HF pose various challenges. Clinicians should recognize the need for dynamic, individual-specific fluid quotas based on risk of shock versus fluid overload. Volume status of patients must be regularly assessed to guide fluid administration, with recognition of the caveats of standard parameters.

## Data Availability

All data generated or analysed during this study are included in this published article.

## Abbreviations

ARDS: Acute respiratory distress syndrome
CKD: Chronic kidney disease
DHF: Dengue haemorrhagic fever
DSS: Dengue shock syndrome
ESRD: End-stage renal disease
FFP: Fresh-frozen plasma
H: Hour
HF: Heart failure
IL: Interleukin
IVC: Inferior vena cava
kg: Kilogram
ml: Millilitres
RL: Ringer lactate
TNF: Tumour necrosis factor
TOF: Tetralogy of Fallot
